# Impact of Intensive Lifestyle Treatment (Diet Plus Exercise) on Endothelial and Vascular Function, Arterial Stiffness and Blood Pressure in Stage 1 Hypertension: Results of the HINTreat Randomized Controlled Trial

**DOI:** 10.3390/nu12051326

**Published:** 2020-05-07

**Authors:** Anastasios Vamvakis, Eugenia Gkaliagkousi, Antonios Lazaridis, Maria G. Grammatikopoulou, Areti Triantafyllou, Barbara Nikolaidou, Nikolaos Koletsos, Panagiota Anyfanti, Christos Tzimos, Pantelis Zebekakis, Stella Douma

**Affiliations:** 1 Third Department of Internal Medicine, Papageorgiou Hospital, Aristotle University of Thessaloniki, GR56403 Thessaloniki, Greece; spanbiol@hotmail.com (A.L.); artriant@hotmail.com (A.T.); barbienn@yahoo.gr (B.N.); nick.koletsos@gmail.com (N.K.); panyfan@hotmail.com (P.A.); sdouma@auth.gr (S.D.); 2 Department of Nutritional Sciences & Dietetics, Faculty of Health Sciences, International Hellenic University, Alexander Campus, Sindos, PO Box 141, GR57400 Thessaloniki, Greece; maria@ihu.gr; 3 Northern Greece Statistics Directorate, Hellenic Statistical Authority, 218 Delfon Str, GR54646 Thessaloniki, Greece; ctzimos@gmail.com; 4 Division of Endocrinology and Metabolism and Diabetes Center, First Department of Internal Medicine, Medical School, AHEPA University Hospital, Aristotle University of Thessaloniki, GR54621 Thessaloniki, Greece; pzempeka@auth.gr

**Keywords:** hypertension, asymmetric dimethylarginine, dietary inflammatory index, endothelial dysfunction, inflammation, medical nutrition therapy

## Abstract

Lifestyle modification is an important component of essential hypertension (EH) therapy. The aim of the Hypertension Intensive Nutrition Treatment (HINTreat) parallel, randomized controlled trial was to examine the effect of a 6-month intensive lifestyle treatment (ILT) (diet plus exercise with monthly visits) compared to the usual care. A total of 76 adults with stage 1 EH were randomized (38 in each group). Dietary analysis, anthropometry, physical activity, biochemical and urine profile, blood pressure (BP), asymmetric dimethylarginine (ADMA), central hemodynamics, β-stiffness index and carotid intima media-thickness were evaluated. The dietary inflammatory index (DII) was calculated for each participant from the intake of 29 nutrients/food components. At the end of the trial, participants in the ILT group reduced their 24h urinary Na excretion (*p* ≤ 0.001), daytime systolic BP (*p* ≤ 0.048) and mean carotid β-stiffness index (*p* ≤ 0.005) and ameliorated their lipidemic profile compared to the standard care. Univariate analysis for the total sample showed a strong association between DII and ADMA levels (β = 0.089, *p* ≤ 0.01). ILT is effective in improving the inflammatory components of the diet and selected cardiometabolic parameters, including arterial stiffness.

## 1. Introduction

Essential hypertension (EH) consists of a pivotal cardiovascular disease (CVD) risk factor, directly associated with a higher incidence of stroke and myocardial infarction, thus rendering EH a major public health concern [1,2]. Endothelial dysfunction is the hallmark of EH and CVD pathophysiology, preceding atherosclerosis, while contributing significantly to the subclinical target organ damage [3,4]. It reflects the loss of the vasodilating properties of the vascular bed, mainly due to a reduced nitric oxide availability, leading to the expression of a pro-thrombotic, pro-inflammatory and also pro-atherosclerotic vasculature phenotype [5]. Among all biomarkers of endothelial dysfunction, endothelial microparticles (EMPs) and asymmetrical dimethylarginine (ADMA) are considered the most robust ones [4]. Micro- and macrovascular dysfunction are both impaired early during the course of EH, entering in a vicious cycle of interaction. Inside this loop, sustaining high blood pressure (BP) inevitably leads to hypertension-mediated target organ damage, which in turn aggregates BP [6]. Arterial stiffness reflects macrovascular function and is mainly affected by ageing and elevated BP [7]. Emerging evidence indicates that subclinical chronic inflammation plays an important role in the pathogenesis, as well as in both micro- and macrovascular dysfunction, and in the target-organ damage [3,8]. Thus, specific biomarkers including C-reactive protein (CRP), interleukins and cytokines (IL-1β, IL-4, IL-6, IL-10, TNF-α) have been extensively examined over the past decade and have been shown to be increased among patients with CVD [9].

Compared to brachial BP, central systolic BP (SBP) is strongly and more closely related to target organ damage and CVD events [10]. Its predictive value has been shown in different patient populations including older adults [11], patients with end-stage renal disease [12] and healthy individuals [13]. In addition, it has been shown that central pulse pressure (PP) has an independent, predictive value and may better predict all-cause mortality, as compared to 24 h SBP [14]. Furthermore, carotid artery stiffening consists of a surrogate marker of macrovascular damage, greatly related to the incident of stroke and CVD mortality [15].

Lifestyle modification plays a key role in the treatment of EH by decreasing the levels of BP [16,17,18], in some cases delaying the onset of pharmacological treatment among patients at stage I EH of low CVD risk [19] and importantly by improving the cardiometabolic profile of patients [20]. In EH, attaining a healthy diet remains the hallmark of non-pharmacological treatment [21]. Nutrition is a strong inflammation modulator, as the effect of both foods and individual nutrients fluctuates between the pro-inflammatory and anti-inflammatory range [22]. The anti-inflammatory nature of a diet can be determined using a variety of tools, including the dietary inflammatory index (DII) [23]. The DII has been associated with a greater risk for developing CVD and metabolic syndrome, the presence of sub-clinical atherosclerosis, as well as with a plethora of inflammatory markers [24,25,26]. Today, the number of interventional studies evaluating changes in the DII in relation to cardiometabolic outcomes [27,28] is limited, with the majority of research being of cross-sectional design. Additionally, the literature is lacking evidence linking DII and ADMA, a reliable marker of endothelial dysfunction.

The Hypertension Intensive Nutrition Treatment (HINTreat) trial was designed with the aim of investigating the effects of a six-month intensive lifestyle treatment (ILT) versus standard care, among patients with stage Ι ΕH.

## 2. Materials and Methods

### 2.1. Ethical Clearance and Protocol Registry

The HINTreat was a prospective, randomized, single-blind, parallel trial performed in Thessaloniki, Greece. Ethical clearance was obtained from the Ethical Committee of the Aristotle University of Thessaloniki’s Medical School, in accordance with the principles of the Helsinki declaration. The protocol was registered at the World Health Organization (WHO) affiliated trial registries (IRCT20200307046715N1). The study was carried out from the year 2016 to 2019, among outpatients of the Hypertension Unit of the 3rd Department of Internal Medicine, situated at Papageorgiou General Hospital, in Thessaloniki, Greece. All participants provided their informed consent prior to participation.

### 2.2. Study Design-Population

A total of 91 adult men and women with early stage I EH according to the European Society of Hypertension (ESH) criteria [29] were recruited from the Hypertension outpatient clinic at Papageorgiou General Hospital. Inclusion criteria involved: (1) untreated stage I EH, (2) age > 18 years old and (3) agreement to participate. Exclusion criteria were: (1) diagnosis of secondary hypertension; (2) diabetes mellitus; (3) CVD (including history of coronary heart disease as evidenced by coronary angiography or a positive treadmill test, myocardial infarction, revascularization procedure, or stroke); (4) any known inflammatory condition; (5) malignancy; (6) or other significant comorbidities affecting arterial BP; (7) treatment with antihypertensive agents or other significant medication, including diuretics. Patients with either white coat or masked hypertension were identified via ambulatory BP measurements (ABPM).

Patients were divided into two groups. In the first group participants acted as controls, receiving standard advise concerning lifestyle modifications, according to the ESH guidelines [29]. Standard advise included two basic sentences reporting the ESH recommendations [29] for diet and exercise, including the reduction of salt intake and the need for increased physical activity (PA), provided once at the start of the trial, without any further details or educational sessions provided throughout the six-month period. In parallel, controls did not receive a personalized dietary schedule. The second group formed the intervention group, receiving ILT concerning nutrition modification and exercise, again based on the ESH guidelines [29], however, in greater detail and duration as compared to the usual care participants. In particular, at baseline, the ILT group received an hourly one-on-one nutrition education session, stressing every point raised within the ESH lifestyle recommendations [29], with a registered dietitian (A.V.) and a personalized diet plan. Emphasis was given to the dietary changes needed to be adhered, including increased fruit and vegetable consumption, reduced salt intake, weight loss and the need for regular physical activity (PA). Thus, although both groups were informed of the ESH lifestyle recommendations [29] for the controls, this did not last more than 5 min, whereas for the ILT participants, detailed hourly sessions were repeated every month throughout the trial.

**Figure 1:**
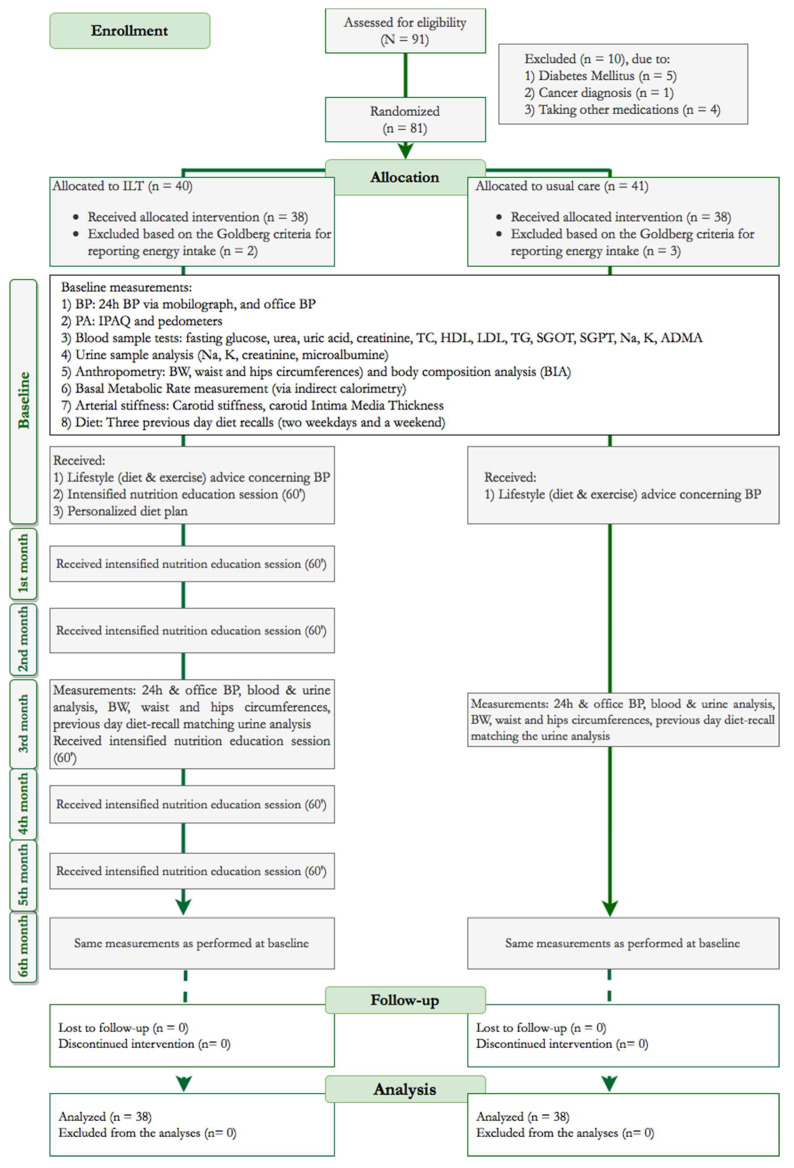
CONSORT diagram of the study’s process. *ADMA*, asymmetric dimethylarginine; *BIA*, bioelectrical impedance analysis; *BP*, blood pressure; *BW*, body weight; *HDL*, high-density lipoprotein; *ILT*, intensive lifestyle treatment; *IPAQ*, international physical activity questionnaire; *LDL*, low-density lipoprotein; *PA*, physical activity; *TC*, total cholesterol; *TG*, triglycerides.

At baseline, a variety of anthropometric, dietary and clinical data were collected for all participants, as detailed in Figure 1.

### 2.3. Intervention Particularities and Frequency

All participants visited the hypertension unit at 3 and 6 months after treatment allocation, for regular BP measurements and assessment of the degree of adherence to the initial advice provided. Compared to the usual care group, intensive treatment patients were supported, with monthly personalized nutrition education (A.V.), lasting for one hour each, throughout the study period (Figure 1).

All visits were performed in early morning hours, after an overnight fast, with participants having avoided intense PA for the 24 h prior. All patients undertook a brief self-affirmation manipulation in order to reduce their defensive biases, better accept the health information and buffer the effect of stress. Additionally, self-affirmation manipulation was expected to increase message acceptance in both groups [30,31]. All baseline measurements were finished before any type of lifestyle modification advices to the patients. Baseline characteristics of the participants in each group are detailed in Table 1.

### 2.4. Randomization and Masking

Randomization of participants to either the intensive lifestyle intervention treatment or the usual care group was performed on blocks of 1, using the StatTrek.com website. Investigators and statisticians were all aware of the allocation at every step of the progress. Participants were blinded as per the allocation or the existence of a parallel group.

### 2.5. Anthropometry

Anthropometric measurements were taken by an International Society for the Advancement of Kinanthropometry (ISAK) Level II certified anthropometrist (A.V.), with patients wearing light clothing, according to the ISAK recommendations [32]. Body weight (BW) was measured with a digital scale (SECA 813, SECA Group, Hamburg, Germany) with a 0.01 kg precision. Height was measured using a wall-mounted stadiometer (SECA 216, SECA Group, Hamburg, Germany), with a 1 mm precision. Body Mass Index (BMI) was calculated for each participant by dividing body weight in kilograms with the square of height in meters.

The Resting Energy Expenditure (REE) of each participant was estimated during morning hours, via indirect calorimetry and the use of a metabolic analyzer, in accordance to the manufacturer’s guidelines (Breezing Pro, Breezing, Tempe AZ, USA) [33,34].

Nutrition data were collected using repeated previous 24 h diet recalls (three at each time-point) for each participant (consisting of two weekdays, and one weekend day) [35], at baseline and the end of the six month intervention for both groups (Figure 1). The first day of the diet-recalls coincided with the first urine analysis sample day. In parallel, one previous day 24 h diet recall was collected for all participants at 3- and 6-months post-intervention, matching the urine sample analysis day. Given that previous 24 h recalls have limitations, the median of the three recorded days in each time-point (baseline or end of intervention) was used to assess the usual intake of participants, to increase validity of the dietary assessment method [36]. The Food Processor dietary analysis software (ESHA, Portland OR, USA) was used for the dietary analyses, complemented with Greek recipes [37].

Goldberg’s energy intake reporting cut-offs were applied to verify adequacy in energy reporting and classify participants as under-reporters, acceptable reporters and over-reporters [38]. Under and over-reporters were excluded from the sample (Figure 1).

### 2.6. Energy Expenditure and Dietary Assessment

The diet’s inflammatory potential was estimated with the dietary inflammatory index (DII) [23], computed from the median of the 24 h diet recalls. The DII was calculated according to the median reported consumption of 29 items for each participant, including the following: energy, protein, carbohydrate, total fat, cholesterol, trans fats, mono-unsaturated fatty acids (MUFA), n-3 and n-6 fatty acids, poly-unsaturated fatty acids (PUFA), alcohol, vitamin B_12_, vitamin B_6_, β-carotene, caffeine, fiber, folic acid, niacin, riboflavin, saturated fatty acids (SFA), thiamin, vitamins (A, C, D and E), iron, zinc, selenium and magnesium. The dietary intake for each of the 29 parameters was adjusted against a reference global intake and divided by the standard deviation [23]. In order to calculate each patient’s DII, each nutrient’s specific contribution to the DII was calculated by each parameter’s centered proportion, multiplied by the respective parameter’s specific inflammatory effect score. Thereafter, individual nutrient scores were pooled to calculate the total DII score of each patient, based on the 29 aforementioned nutrients.

Given that the score of each dietary index is greatly dependent on participants’ energy intake, crude DII was regressed on the energy intake, producing an energy adjusted DII score (DII_adj_), based on the residual method [39,40].

Since dietary sodium (Na) intake is not a component of the DII, but consumption is highly related to BP levels [41], the median intake of the three previous 24 h recalls was used to calculate the crude reported intake of participants. In parallel, dietary Na intake was also assessed with the correction method proposed by Mercado [42], assuming that 90% of the excreted urinary Na corresponds to a more accurate dietary Na intake.

Salt sensitivity [43] was not assessed among participants, as the protocol is demanding, and this might reduce the number of participants willing to participate in the present trial.

### 2.7. Assessment of Physical Activity Levels

Self-reported PA was recorded using the International Physical Activity Questionnaire (IPAQ) [44]. The IPAQ collects information concerning the intensity (vigorous, moderate, walking or sitting) and the duration and frequency (average time, number of days) of PA in various categories (work, transportation, sports and house chores). The PA of each category and intensity was expressed in metabolic equivalents (METs) and summed for the estimation of the weekly PA. MET intensity tiers were classified IPAQ questions for either vigorous (8 METs) or moderate PA (4 METs) and walking (3.3 METs) [44]. Objectively reported levels of PA were recorded using pedometers (Omron Jog Style HJA 300-EK, Omron Co, Kyoto, Japan) for a 7-day period (same as the self-reported PA). Data from daily average step counts (from the total 7-days period) were converted to PA categories into low (<7500 steps/day), moderate (7500–10,000 steps/daily), or high PA (>10,000 steps/24 h) [45].

### 2.8. Biochemical Markers and Urinary Analysis

Morning fasting blood samples were collected for biochemical (glucose, SGOT, SGPT, sodium, potassium, urea, creatinine and uric acid) and lipidemic profile analysis, including total cholesterol (TC), triglycerides (TG), low-density lipoprotein (LDL) and high-density lipoprotein (HDL). Additionally, blood samples of 20 mL were collected from each patient, serum was separated, and samples were stored at −80 °C. For each participant, 24 h urinary samples were analyzed for Na (Sodium) and K (Potassium) excretion. In order to reduce the Hawthorne effect [46], during the urine collection days, participants were advised to adhere to their usual dietary habits, without any efforts to ameliorate their diet further.

### 2.9. Blood Pressure Measurements

Office BP was measured with participants in a seated position after 10 min of rest, in the arm exhibiting the highest BP value, using a validated oscillometric device (Microlife Exact BP, Microlif AG, Widnau, Switzerland). Both systolic (SBP) and diastolic blood pressure (DBP) measurements were recorded. For office BP, the mean of the second and the third values of three consecutive measurements was recorded, with 2 min intervals between each measurement. Branchial and aortic ABPM measurements were taken from the upper non-dominant arm using a Mobil-O-Graph Holter device (I.E.M. GmbH, Stolberg, Germany), for which at least 70% of the readings were successful [47,48]. EH was diagnosed when office BP > 140/90 mmHg, and when brachial ABPM > 135/85 mmHg, on average daytime, according to the ESH guidelines [29]. Additionally, the protocol for ABPM was set at three BP measurements per hour during daytime (07:00–22:59), and two/hourly during night-time (23:00–06:59).

### 2.10. Endothelial Function

A commercially available competitive enzyme-linked immunosorbent assay (ELISA) kit for ADMA (DLD Diagnostika GmbH, Hamburg, Germany), with a sensitivity 0.05 μmol/L, was used to assess endothelial dysfunction in all individuals. After blood sampling, serum was separated and stored at −80 °C until further analysis. All assays were performed by the same investigator.

### 2.11. Vasculr Function: Carotid Measurements and Central Hemodynamics

Vascular assessments included measurements of aortic central BP with the Mobil-O-Graph 2 device (Numed Healthcare, Sheffield, UK). Subclinical atherosclerosis in the carotid arteries was evaluated with assessment of carotid intima-media thickness (cIMT). Longitudinal images of the common carotid arteries were taken in the supine position using an ultrasound machine (Aloka Pro Sound A7, Ultrasound System, Tokyo, Japan). cIMT of the left and right common carotid arteries was determined by the mean of two consecutive measurements in the far wall of the distal, 10 mm for each artery [49].

Carotid arterial stiffness was estimated by measuring carotid stiffness (β-stiffness index) from both the left and the right carotid arteries, using a high definition echo-tracking system (Aloka Pro Sound A7, Ultrasound System, Tokyo, Japan). The β-stiffness index was calculated as the ratio of the natural logarithm of SBP/DBP, to the relative change (Δ) in diameter [50]. Before the measurements, subjects lay down in a supine position and stay rested for approximately 10–15 min. At least 9 beats are needed to record a representative waveform, and the average of the two consecutive measurements per carotid was used to calculate the final β-stiffness index values [51].

### 2.12. Primary and Secondary Outcomes of Interest

The primary outcomes of interest included changes in brachial office, ambulatory BP and in central 24 h BP after 6 months of follow-up. Changes in indices of arterial stiffness specifically in carotid stiffness and central hemodynamics were also included. Other markers of subclinical atherosclerosis such as cIMT and lipid profile (TC, TG, HDL, LDL) were also determined for the same period of follow up.

Secondary outcomes involved changes (Δ) in body weight, BMI, waist and hip circumferences, levels of PA, DII, as well as, urinary Na and K losses.

### 2.13. Treatment Compliance and Follow-Up

Treatment compliance was assessed in the intensive treatment group using the previous day diet-recalls and the urine analysis (for Na restriction). Adherence to the physical activity guidelines was assessed using both the IPAQ and the pedometer records.

Although the duration of the intervention lasted for a total of 6 months, patients were followed for an additional 6-months after the completion of the intervention. However, these data will be analyzed separately.

### 2.14. Statistical Analyses

To rule out selection bias, we compared the variables between participants of both groups. To estimate the effect of intervention, a *t*-test was performed between two groups. We also evaluated the results of the 6-month intervention compared to the baseline. Normal distribution was assessed either with the Kolmogorov—Smirnov or with the Shapiro—Wilk test. Non-normally distributed variables were tested using the Mann–Whitney U test. Parametric tests were used among normally distributed variables, with paired *t*-tests being used to assess baseline and end-of treatment differences and independent samples *t*-tests applied to investigate differences between groups. Nevertheless, mean ± standard deviation (SD) values or *n* with the respective % were used to present data in a uniform manner and avoid reader confusion. The Pearson’s correlation was used to assess the relationship between the two different PA methods used.

Linear regression models were also computed to examine whether differences in ADMA levels (continuous) were related to relevant changes in the DII (continuous), post-intervention.

All tests were two-sided at a significance level of 5%. The statistical analyses were performed using Statistical Package for Social Sciences (SPSS) version 25.0 (IBM, Armonk NY, USA). Due to the plethora of recorded parameters and outcomes, the majority of secondary outcomes will be presented in a subsequent publication. A *per protocol* analysis was performed.

## 3. Results

### 3.1. Drop-Outs

Out of 81 patients randomized in total (Figure 1), two from the ILT group and three from the controls were excluded from the analyses for having extreme energy intake records according to the Goldberg criteria for adequate energy reporting. Thus, a total of 38 patients formed the final samples in each group.

### 3.2. Na Intake and Blood Pressure Measurements

At the end of treatment, urinary Na excretion was significantly lower in the ILT as compared to the baseline and the control group (*p* ≤ 0.001 for both). Reported dietary Na intake did not differ; however, when dietary Na intake was calculated from the urinary Na excretion, a significant reduction was noted in the ILT compared to the baseline as well as a significantly lower intake as compared to the usual care participants, at the end of the treatment (*p* ≤ 0.001 for both).

Among patients in the ILT group, post-treatment SBP and DBP, based on both office and ABPM measurements, expressed either as 24 h or daytime values, were significantly improved compared to the baseline and compared to the control group (Table 2).

### 3.3. Blood and Urine Analyses

In the ILT group, a significant reduction was noted in the plasma levels of TC, TG and LDL, as well as in the urinary Na losses at the end of the treatment, as compared to the baseline measurements (*p* ≤ 0.001 for all), and to the controls (*p* ≤ 0.001 for all) (Table 2). No differences were noted in the reported Na intake based on the previous 24h recalls, however, Na intake calculated by the urinary Na excretion was significantly reduced in the ILT as compared to the baseline and to the controls (*p* ≤ 0.001 for both).

### 3.4. Endothelial and Vascular Function

Six months of ILT were efficient in improving ADMA levels (*p* ≤ 0.001) (Table 2). Improved 24 h central SPB and DBP were also noted among ILT participants compared to the baseline (*p* ≤ 0.002 and *p* ≤ 0.001, respectively).

When compared to the standard care group, ILT patients demonstrated improved ADMA levels (*p* ≤ 0.001), cIMT (*p* ≤ 0.037) and carotid stiffness expressed as the β-stiffness index (*p* ≤ 0.05). SBP and DBP were also improved according to the 24 h measurements (*p* ≤ 0.02 and *p* ≤ 0.011, respectively) and the daytime records (*p* ≤ 0.001 and *p* ≤ 0.012, for SBP and DBP, respectively).

### 3.5. Nutritional Assessment and Physical Activity Levels

At the end of the intervention BMI was significantly reduced in the ILT group compared to the baseline (27.5 ± 4.3 vs. 29.7 ± 5.1 kg/m^2^, *p* ≤ 0.045). The difference in baseline and end-of-treatment BMI was not significant among controls (28.8 ± 4.1 vs. 28.7 ± 4.0 kg/m^2^ during the start and end of the trial, respectively, *p* ≤ 0.915).

No improvements were observed in the physical activity volume of participants in either group. In further detail, based on the pedometer measured PA, the volume of PA at baseline was 869.5 ± 461.6 MET-min/week in the ILT and 1103.1 ± 572.1 MET-min/week in the standard care group respectively, whereas at the end of treatment, PA reached 1032.7 ± 632.8 MET-min/week among ILT participants and 1007.4 ± 533.5 MET-min/week among controls. According to the IPAQ PA, at baseline, ILT participants reported a PA volume of 1516.7 ± 1333.2 MET-min/week and controls a mean PA reaching 1269.8 ± 1116.3 MET-min/week. Six months after the trial’s initiation, participants in the ILT reported a mean PA volume of 2173.9 ± 2052.8 met-min/week, whereas controls had a respective PA of 1882.9 ± 2290.1 MET-min/week. When agreement between the two methods was tested, weak correlations were exhibited at the start (r = 0.254 and *p* ≤ 0.027) and the end of treatment (r = 0.256 and *p* ≤ 0.026).

According to the dietary analysis, the DII was improved in both groups compared to the baseline (*p* ≤ 0.001 for both), even after adjustment for the energy intake of participants (Table 3).

Between groups, at the end of treatment, those allocated in the ILT exhibited an improved DII as compared to the controls (*p* ≤ 0.001) (Table 3). Among the pro-inflammatory nutrients at the same time-point, energy and fat intake were significantly lower in the ILT as compared to the usual case group. On the other hand, a higher intake of many anti-inflammatory nutrients was noted in the ILT at 6-months in comparison to the usual care, including n-3 and n-6 fatty acids, MUFA, fiber, vitamins (A, B_6_, C, D, E), β-carrotene, ribovlavin, folic acid, thiamin, niacin, as well as Mg, Se and Zn.

Within groups, comparisons examining the dietary intake at the start and end of treatment (Table 3) revealed a significantly reduced DII among ILT participants (both crude and adjusted to the energy intake). In the control group, only the DII adjusted to the energy intake was reduced as compared to the baseline. However, the mean DII of ILT patients reached anti-inflammatory levels (DII < 0), whereas in the usual care group, it remained at pro-inflammatory numbers (DII > 0). Energy intake was reduced only among participants following the ILT regime, without any significant difference being recorded among controls. Six months of ILT induced significant improvements in the dietary intake of selected pro-inflammatory nutrients, including energy, cholesterol, SFA and trans fats, as well as an increased consumption of anti-inflammatory nutrients like the n-3 and n-6 fatty acids, fiber, vitamins (A, B_6_, B_12_, C and D), riboflavin, folic acid, niacin, thiamin, β-carotene, Mg, Se and Zn. Among controls, only the intake of SFA, MUFA and vitamin A were improved after six months.

### 3.6. Prediction of Change in ADMA Levels According to the DII Score

The value of R^2^ (0.517) for the differences of DII adjusted to the energy intake of participants indicates that variations in the ADMA levels can be explained by the DII in 52% of the cases (Table 4). In the total sample, improvement (reduction) of the DII adjusted to the energy intake of participants by 1 unit was associated with a 0.9 μmol/L change in ADMA. In the ILT group, improvement of the crude DII by 1 unit at 6 months was associated with a 0.7 μmol/L change in ADMA.

## 4. Discussion

The HINTreat results support the hypothesis that a 6-month intensive consultation by dieticians is effective in improving CVD risk factors among patients with early stage I EH, as compared to the standard care. More specifically, brachial and central BP levels, carotid stiffness and endothelial function were all improved after ILT for six months. In addition, thorough guidance for lifestyle modification by a professional led to improved DII, lipid profile and a reduced BMI and urinary Na excretion. Of note, dietary changes aiming to reduce the pro-inflammatory components of the diet were successful in predicting improvement of endothelial function.

The influence of diet on the CVD profile and in particular BP is undeniable. High Na intake can contribute to the development of hypertension, while on the other hand, Na excretion, as a surrogate marker of Na intake, is highly associated with BP levels [41]. Increased dietary Na induces subsequent increases in the retention of Na, augmenting venous tone and central blood volume, propelling the development of hypertension [52,53]. According to the WHO, most people consume on average 9–12 g of salt every day [54]. Dietary interventions have proved successful in reducing daily Na intake (from 11.5 g to 3.8 g), after at least 7 days of intensive treatment [55]. In the present study, 24 h urine analysis revealed a significant reduction in Na excretion. Possibly, the repeated empowering offered in intensive dietary treatment might help mitigate Na intake and increase the consumption of “healthier” foods, including fruit and vegetables [56]. According to the VALNORM trial [57], intensive education is more beneficial as compared to providing basic support. Additionally, it may well be easier for patients to understand hypertension-related dietary modifications, as both salt restriction and identification and avoidance of salty foods is relatively easier as compared to other Medical Nutrition Therapy (MNT) constituents.

Apart from BP, the lipid profile of participants was also significantly improved following the ILT, as outlined in several previous RCTs [58,59] and meta-analyses [60]. Overall, the influence of a dietary intervention to reduce BP appears to exert beyond the absolute BP indices, ameliorating many additional CVD contributors, including serum TC, LDL, HDL and TG levels.

In parallel to BP however, indices of vascular stiffness were also improved. Not only were 24 h central BP measurements reduced after the ILT, but also carotid stiffness was decreased, representing more local hemodynamics at the carotid artery site. These findings are in keeping with the existing knowledge as a variety of dietary factors have been proved to improve vascular function, including weight loss [61,62,63,64], consumption of fermented dairy products, salt restriction, as well as increased fish oil, soy isoflavones and n 3 fatty acids intake [64,65,66]. More than that in the present study, a regression of atherogenesis was noted, as this is depicted by the measurement of cIMT.

Importantly, although individuals had early stage I EH, a reduction in ADMA levels was noted after six months of ILT. Increased levels of ADMA have been observed in EH even from the early course of the disease preceding clinically evident atherosclerosis and cardiovascular complications and tend to correlate with arterial stiffness [4,67]. The finding is of great importance as it indicates that early intensive lifestyle modification may reverse and even delay the initialized vascular damage in EH.

In the present trial, ILT was efficient in improving the diet of participants, in relation to the inflammatory dietary constituents. As a result, the DII was significantly improved post-intervention, either as crude or adjusted to the energy intake, indicating that one-on-one nutrition education sessions can improve the diet of hypertensive patients more than providing standard nutritional advice at diagnosis. In particular, the consumption of several anti-inflammatory components of the diet was increased (zinc, selenium, magnesium, etc.), whereas the intake of pro-inflammatory ones was decreased (trans fats, cholesterol, etc.) To date, research has shown that longitudinal low grade inflammation is a factor propelling the progression of vascular abnormalities, including arterial stiffness, elevating hypertension [68,69]. As such, many anti-inflammatory foods and nutrients have been suggested as BP decreasing modulators [70,71]. Primary research has associated increased DII with an elevated risk for developing CVD [72,73], with one meta-analysis verifying this finding, while also revealing a propensity for all-cause, cardiovascular and cancer-related mortality in patients demonstrating increased DII scores [74].

Concerning Na intake of participants, our study agrees with previous research, regarding the lack of validity of self-reported Na intake [75]. According to the results, it appears that the reported intake differed from the measured urinary Na excretion, and this is why measuring urinary Na is considered as the gold standard for estimating Na intake [76,77]. Furthermore, it appears that intensive counseling can reduce the Na intake of hypertensive patients, whereas, on the other hand, simple reporting of the recommendations does not appear to be an adequate intervention inducing significant consumption changes.

The role of an experienced, registered dietitian is also highlighted by the results herein. ILT participants were receiving MNT and one-on-one nutrition education sessions every month and appeared to have benefited the most, as compared to those receiving standard dietary advice once. The importance of having MNT delivered by dietitians has already been reported in the literature [78,79,80], as they are the most competent in the field, leading to a more effective treatment of the underlying disease [81,82].

On the other hand, research on the relationship between ADMA and diet is scarce. Interestingly, herein, ADMA was greatly dependent on the DII content of the diet. Chronic salt loading has been suggested to increase plasma ADMA and BP [83]. According to the literature, ADMA is improved following high-fiber diets [84] or increased vegetables and tea intake [85]. Indeed, the ILT resulted in an increased fiber intake, which might have contributed to mitigating ADMA levels among participants.

The results also revealed a reduction in patient BMI 6 months after initiating the ILT. Based on the latest guidelines for EH [86], reducing BMI is crucial when hypertensive patients are concerned, as 1 kg of body weight loss results in a 1 mmHg BP reduction [87]. Crimarco and associates showed that participation in a 12 month ILT improved body weight, reduced BP and promoted selective anti-inflammatory dietary components [28].

Nevertheless, the present RCT is not without limitations. The number of outcomes increased the difficulty in presenting all results simultaneously, whereas in parallel, the sample consisted of patients with stage I EH only. It is unclear if the present findings can be exerted to patients at higher stages of hypertension or those with comorbidities. Moreover, given that the intervention consisted of both dietary and exercise protocols, it is possible that people with moving difficulties might not experience the same benefits as the ones participating herein. Finally, salt sensitivity [43] of participants was not assessed and it is likely that if measured, the results might have been different. The strong points of the study include the valid measurement of Na intake via urine samples [75]. Concerning the dietary intake assessment, given that previous 24 h recalls have inherited limitations, the usual intake of participants was calculated herein [36,88]. In parallel, the meticulous exclusion of participants who were low or high energy reporters further increased the validity of the results.

## 5. Conclusions

In summary, the HINTreat study showed that adhering to an ILT can improve BP, vascular and endothelial function among patients with early stage I EH. Moreover, delivery of MNT in an intensive frequency manner and by a registered dietitian appears to be more beneficial as compared to the usual care. Additionally, adhering to the dietary recommendations for EH improves the DII score of the diet and might ameliorate ADMA levels and endothelial dysfunction. Future RCTs need to focus more on the impact of lifestyle modification, involving nutrition specialists and the manipulation of pro-inflammatory components on BP, arterial stiffness and endothelial dysfunction in order to mediate the global burden of CVD.

## Figures and Tables

**Table 1:** Patient characteristics at baseline.

		ILT (*n* = 38)	Usual Care (*n* = 38)	*p* Value
Men/women (*n*, %)		16/22 (42.1%/57.9%)	24/14 (63.2%/36.8%)	NS ^χ^
Age (years)		48.4 ± 10.2	48.4 ± 11.3	NS
Body weight (kg)		86.3 ± 16.7	84.3 ± 13.8	NS
BMI (kg/m^2^)		29.7 ± 5.1	28.8 ± 4.1	NS
Overweight ^†^/Obese ^‡^ (*n*, %)		20/14 (53%/37%)	13/16 (34%/42%)	NS ^χ^
Waist circumference (cm)	*Men*	107.3 ± 11.1	102.1 ± 11.1	NS
	*Women*	90.8 ± 11.3	93.6 ± 11.9	NS
REE (kcal/24 h)	*Men*	1898 ± 381	1888 ± 313	NS
	*Women*	1596 ± 255	1663 ± 284	NS
Smoking (*n*/%)		12 (32%)	15 (40%)	NS ^χ^
Daytime SBP (mmHg)		139.9 ± 9.7	136.9 ± 7.4	NS ^M^
Daytime DBP (mmHg)		92.3 ± 9.5	89.5 ± 7.2	NS

^χ^ Tested with the chi-square test. ^M^ Tested with the Mann–Whitney U test. ^†^ 25 ≤ BMI < 29.99 kg/m^2^. ^‡^ BMI ≥ 30 kg/m^2^. *BMI*, Body Mass Index; *DBP*, Diastolic Blood Pressure; *ILT*, Intensive Lifestyle Treatment; *NS*, Not Significant; *REE*, Resting Energy Expenditure; *SBP*, Systolic Blood Pressure.

**Table 2:** Blood and urine analyses, endothelial function, central hemodynamics and arterial stiffness at baseline and post-treatment in both groups.

		ILT (*n* = 38)		Usual Care (*n* = 38)	
Variables		Baseline	End of Treatment	Baseline	End of Treatment
Serum levels	Glucose (mg/dL)	91.8 ± 10.5	95.8 ± 11.0 ^M^	93.1 ± 10.2	93.9 ± 11.6 ^M^
	TC (mg/dL)	213.6 ± 39.7	177 ± 20.3 ***^†††M^	210.3 ± 37.1	207.7 ± 38.8 ^M^
	TG (mg/dL)	129.6 ± 28.7	95.3 ± 39.5 ***^†M^	141.6 ± 25.5	119.5 ± 55.9 *^M^
	HDL (mg/dL)	46.7 ± 9.1	48.6 ± 10.5	45.2 ± 10.3	45.8 ± 10.1
	LDL (mg/dL)	147.5 ± 36.9	119 ± 20.9 ***^††^	138.5 ± 33.7	136 ± 30.5
Na	Urinary Na (mmol/24 h)	139.1 ± 42.4	87.6 ± 24.2 ***^†††^	152.4 ± 74.7	153.8 ± 75.4
	Reported dietary Na intake ^‡^ (mmol/24 h)	154.8 ± 55.6	127.4 ± 43.7	176.2 ± 38.1	154.5 ± 33.8
	Dietary Na ^#^ intake based on urinary loss (mmol/24 h)	153 ± 46.6	96.3 ± 26.6 ***^†††^	167.6 ± 82.2	169.2 ± 82.9
***Blood pressure:***					
Office	SBP (mmHg)	142.8 ± 4.1	123.3 ± 8.9 ***^†††^^M^	140.6 ± 6.6	135.7 ± 9.5 *^M^
	DBP (mmHg)	90.9 ± 9.1	82.2 ± 7.6 ***^†^^M^	88.5 ± 7.8	86.4 ± 9.1 ^M^
ABPM	SBP 24 h (mmHg)	135.1 ± 9.6	124.7 ± 10.2 ***^††^	133.5 ± 7.4	134.1 ± 12.4
	DBP 24 h (mmHg)	88.8 ± 8.5	81.3 ± 7.1 ***^††^^M^	86.4 ± 7.1	86.3 ± 8.2 ^M^
	SBP daytime (mmHg)	139.9 ± 9.7	127.7 ± 9.8 ***^†††^	136.9 ± 7.4	137.5 ± 12.8
	DBP daytime (mmHg)	92.3 ± 9.5	84.7 ± 7.4 ***^†^	89.5 ± 7.2	89.3 ± 8.3
***Endothelial function:***					
ADMA (μmol/L)		1.02 ± 0.12	0.59 ± 0.12 ***^†††M^	0.97 ± 0.34	1 ± 0.32 ^M^
***Central hemodynamics:***					
	SBPc 24 h (mmHg)	136.6 ± 10.2	128 ± 12.6 **^††^	134.9 ± 9.1	136.9 ± 11.5
	DBPc 24 h (mmHg)	90.3 ± 8.3	83.1 ± 7.2 ***^†^	87.6 ± 7.4	87.8 ± 8.4
	PP^c^ 24 h (mmHg)	45.8 ± 7.7	43.4 ± 7.4 ^†M^	47.1 ± 7.7	48 ± 8.9 ^M^
***Carotid Stiffness and subclinical atherosclerosis:***					
US	Mean cIMT (mm)	0.6 ± 0.07	0.58 ± 0.08 ^†^	0.62 ± 0.1	0.63 ± 0.1
	Mean β-stiffness index	8.4 ± 2.3	6.9 ± 1.6 **^††M^	7.7 ± 1.8	8.5 ± 2.8 ^M^

*ABPM*, ambulatory blood pressure monitoring; *ADMA*, asymmetric dimethylarginine; *BP*, Blood pressure; *DBP*, diastolic blood pressure; *HDL*, high-density lipoprotein; c*IMT*, carotid intima media thickness; *ILT*, intensive lifestyle treatment; *LDL*, low-density lipoprotein; *Na*, sodium; *PP*, pulse pressure; *SBP*, systolic blood pressure; *TC*, Total cholesterol; *TG*, triglycerides; *US*, ultrasound. ^M^ Tested with the Mann–Whitney U test. ^c^ Central. ^‡^ Based on the median of the previous 24 h recalls for each participant. ^#^ Assessed by 24 h Na urinary excretion, assuming that 90% of 24 h dietary Na intake is excreted in the urine [42]. * Statistically different compared to the baseline measurements (*** *p* ≤ 0.001, ** *p* ≤ 0.01, * *p* ≤ 0.05). ^†^ Statistically different compared to the usual care, at the same time point (^†††^ *p* ≤ 0.001, ^††^ *p* ≤ 0.01, ^†^ *p* ≤ 0.05).

**Table 3:** Analysis of the dietary intake and components of the dietary inflammatory index (DII) at baseline and post-treatment, for both groups.

	ILT (*n* = 38)		Usual Care (*n* = 38)	
	Baseline	End of Treatment	Baseline	End of Treatment
DII (crude)	5.54 ± 1.15	−0.16 ± 0.95 ***^†††M^	5.21 ± 1.31	4.97 ± 1.63 ^M^
DII_adj_	5.34 ± 0.41	−0.16 ± 0.26 ***^†††^	5.42 ± 0.38	4.97 ± 0.43 ***
***Daily dietary intake (DII Components):***				
Energy Intake (kcal) ^p^	2015.3 ± 416.0	1811.7 ± 302.2 *^†^	2092.5 ± 412.3	1982.2 ± 339
Carbohydrate (g) ^p^	191.5 ± 53.7	174.6 ± 38.5	200 ± 47.8	180.5 ± 46.8
Protein (g) ^p^	69.7 ± 25.9	60.5 ± 18.5	70.1 ± 22.7	68.8 ± 23
Fat (g) ^p^	106.3 ± 28.5	96 ± 16.8 ^††^	113 ± 24.4	108.7 ± 20.9
Cholesterol (mg) ^p^	209.2 ± 128.8	140.6 ± 114.9 *^M^	170.1 ± 94.2	130.6 ± 102.7 ^M^
SFA (g) ^p^	28.3 ± 10.9	20.4 ± 5.6 ***^M^	27.1 ± 10.4	19.8 ± 6.7 ***^M^
Trans fats (g) ^p^	1.9 ± 2.1	1.1 ± 0.4 *^M^	1.8 ± 2.5	1.1 ± 0.6 ^M^
n−3 fatty acids (g) ^a^	0.7 ± 0.2	1.1 ± 0.4 ***^†††M^	0.7 ± 0.3	0.6 ± 0.3 ^M^
n−6 fatty acids (g) ^a^	8.2 ± 3.4	9.7 ± 2.6 *^††M^	8.3 ± 3.7	7.5 ± 3.2 ^M^
MUFA (g) ^a^	55 ± 11.3	52.8 ± 8.4 ^††M^	53.2 ± 15.8	44.8 ± 10.8 **^M^
PUFA (g) ^a^	11.3 ± 3.2	13.3 ± 3.5 *^M^	11.8 ± 3.7	11.4 ± 5 ^M^
Fiber (g) ^a^	16.8 ± 6.9	27.5 ± 7.3 ***^†††M^	17 ± 5.8	17.3 ± 5.6 ^M^
Alcohol (g) ^a^	2 ± 7.5	2.8 ± 8.7 ^M^	0 ± 0	1 ± 4.8 ^M^
Caffeine (g) ^a^	1 ± 0.9	1.1 ± 0.3 ^M^	0.7 ± 0.6	1 ± 0.7 *^M^
Vitamin A (RE) ^a^	478.8 ± 252.1 ^†^	1074.1 ± 388.4 ***^†††M^	710 ± 557.6	489.4 ± 325.5 *^M^
β–carotene (mg) ^a^	0.7 ± 0.8 ^†^	3.4 ± 1 ***^†††^	1.2 ± 1.1	1.6 ± 1.3 ^M^
Vitamin B_6_ (mg) ^a^	1 ± 0.4	1.6 ± 0.4 ***^†††^	1 ± 0.3	1 ± 0.3
Vitamin B_12_ (μg) ^p^	1.9 ± 1.1	1.2 ± 0.8 **^M^	2.1 ± 1.1	1.8 ± 1.5 ^M^
Riboflavin (mg) ^a^	1.2 ± 0.6	1.7 ± 0.7 **^††M^	1.3 ± 0.6	1.2 ± 0.6 ^M^
Folic Acid (μg) ^a^	169.7 ± 95.4	291.7 ± 48.4 ***^†††M^	177.1 ± 84.7	193.8 ± 92.2 ^M^
Niacin (mg) ^a^	12.1 ± 6.7	24.2 ± 10.7 ***^†††M^	13.6 ± 7.6	13.9 ± 7.4 ^M^
Thiamin (mg) ^a^	1.1 ± 0.5	1.5 ± 0.5 ***^††M^	1.3 ± 0.7	1.1 ± 0.5 ^M^
Vitamin C (mg) ^a^	86 ± 70.3	121.6 ± 57.2 *^††M^	76 ± 49.3	79.6 ± 54.3 ^M^
Vitamin D (μg) ^a^	1.3 ± 1.2	3.9 ± 1.7 ***^†††M^	1.6 ± 1.3	1.7 ± 2.2 ^M^
Vitamin E (mg) ^a^	10.4 ± 3	11.5 ± 3.1 ^††M^	10 ± 2.1	9.2 ± 2.9 ^M^
Iron (mg) ^p^	11.3 ± 4.8	10.9 ± 2.7 ^M^	11.9 ± 6.2	12.8 ± 8.3 ^M^
Magnesium (mg) ^a^	193.3 ± 61.2	299.2 ± 43 ***^†††M^	197.9 ± 63	199.2 ± 77.3 ^M^
Selenium (μg) ^a^	44 ± 25.7	67.9 ± 22.6 ***^†††M^	66 ± 70.2	47.1 ± 24.1 ^M^
Zinc (mg) ^a^	7 ± 2.7	8.6 ± 1.7 **^†††M^	7 ± 2.2	6.7 ± 2.1 ^M^

*adj*, adjusted for the energy intake; *DII*, dietary inflammatory index [23]; *ILT*, intensive lifestyle treatment; *MUFA*, mono-unsaturated fatty acids; *PUFA*, poly-unsaturated fatty acids; *RE* Retinol equivalents; *SFA*, saturated fatty acids. ^a^ anti-inflammatory nutrients. ^M^ Mann–Whitney U test (non-parametric). ^p^ pro-inflammatory nutrients. * Statistically different compared to the baseline (*** *p* ≤ 0.001, ** *p* ≤ 0.01, * *p* ≤ 0.05). ^†^ Statistically different compared to the usual care group, at the same time point (^†††^ *p* ≤ 0.001, ^††^ *p* ≤ 0.01, ^†^ *p* ≤ 0.05).

**Table 4:** Univariate linear regression models describing the relationship between Δ ADMA (dependent variable) and Δ DII (independent variable).

Models		*β* Coefficient	95% CI	*p* Value
Total sample (*n* = 76)	Δ DII_adj_	0.089	0.069 to 0.109	≤0.001
	Δ DII	0.090	0.077 to 0.103	≤0.001
ILT (*n* = 38)	Δ DII_adj_	0.012	−0.047 to 0.070	NS
	Δ DII	0.069	0.045 to 0.094	≤0.001

Δ, difference between end of treatment and baseline; *adj*, adjusted to the energy intake; *ADMA*, asymmetric dimethylarginine; *CI*, confidence intervals; *DII*, dietary inflammatory index [23]; *ILT*, intensive lifestyle treatment; *NS*, not significant.
